# Understanding the journeys of patients with an asthma exacerbation requiring urgent therapy at a primary care clinic

**DOI:** 10.1186/s12890-022-02024-9

**Published:** 2022-06-16

**Authors:** Jing Sheng Quek, Wern Ee Tang, Elya Chen, Helen Elizabeth Smith

**Affiliations:** 1 National Healthcare Group Polyclinics, 3 Fusionopolis Link, Nexus@one-north, South Tower, #05-10, Singapore, 138543 Singapore; 2 Lee Kong Chian School of Medicine, Nanyang Technological University Singapore, Clinical Sciences Building, 11 Mandalay Road, Level 18, Singapore, 308232 Singapore

**Keywords:** Asthma exacerbation, Control, Self-management, Delay, Health-seeking behaviour, Education

## Abstract

**Background:**

Asthma is a significant health issue in primary care. We examined the journeys of patients with asthma exacerbations requiring urgent therapy at a primary care clinic in Singapore.

**Methods:**

Face-to-face semi-structured interviews were conducted with patients who received urgent therapy for asthma exacerbation at a primary care clinic. Data collected was used to construct themes.

**Results:**

Fifteen multi-ethnic adult patients were recruited. Participants cited treatment cost, underuse of preventer medication, difficulties attending routine asthma care due to work, and stigma as barriers to asthma control. Reasons for delay in seeking urgent care for asthma were: inability to access medical care out of hours, competing priorities, perception that an exacerbation was ‘not serious enough’, difficulty recognizing symptoms of asthma exacerbation, and being tired or despondent. Participants were triggered to seek care due to failure of reliever inhalers, duration of symptoms, sleep disturbance, inability to work, or advice from others. During an exacerbation, participants often initiated other self-management measures besides using reliever medication. This included over-the-counter medications and non-pharmacological interventions (e.g. drinking water). Of the 15 patients interviewed, only one stepped up preventer inhaler adequately, according to their Asthma Action Plan (AAP).

**Conclusions:**

In caring for patients with asthma, primary care providers should address patients’ asthma self-management skills, such as recognizing symptoms of asthma exacerbations and regular preventer use, and provide clear instructions on how to respond to asthma symptoms (AAP). Minimizing direct (medication and consultation fees) and indirect costs (loss of earnings and adverse impact on employment prospects) are also important considerations.

## Background

Asthma is the most common chronic respiratory condition in Singapore, affecting up to 5% of adults [1] and 20% of children [2, 3]. Asthma-related hospitalization and mortality rates are three times that of other developed countries like the United States [2]. Asthma costs to the individual are high, with average annual costs for the patient ranging from S$214 (US$158) [4] to S$614 (US$454) [5]. There is potential for asthma care to be improved locally.

In Singapore, patients seeking medical attention can choose between publicly-funded primary care clinics (polyclinics), private General Practitioner (GP) clinics, or the Accident and Emergency (A&E) departments of government-subsidized (or restructured) or private hospitals. Consultation fees vary. Polyclinic consultation charges are S$6.90 (US$5.10) to S$13.20 (US$9.80) for Singaporeans, and primary care consultation fees for chronic disease management can be partially offset by MediSave monies (MediSave is a mandatory national medical savings scheme that help individuals set aside part of their income to pay for their personal or approved dependants’ healthcare expenses). In contrast, A&E attendance fees are upwards of S$120 (US$89), which are either borne wholly by patients, or may be partially offset by optional private insurance.

An asthma exacerbation is an episode of progressive increase in breathlessness, cough, wheezing or chest tightness, and decrease in lung function [6]. Adherence to inhaled corticosteroids (ICS) reduces the risk of asthma exacerbations [7]. Local guidelines (2008 Ministry of Health asthma clinical practice guideline [8] and 2020 Agency for Care Effectiveness Clinical Guidance [9]) also recommend providing an Asthma Action Plan (AAP) for all patients to aid in asthma self-management. AAPs reduce hospitalization rates, days off from work or school [10], and risk of fatal asthma exacerbations [11].

In Singapore, patients presenting to polyclinics with an acute asthma exacerbation are treated with inhaled bronchodilator therapy: short-acting beta-agonist (SABA), with or without a short-acting muscarinic antagonist (SAMA), administered either via nebulization or metered-dose inhaler with spacer (‘dry nebulizer’ therapy). For the purposes of this study, both forms of inhaled bronchodilator therapy were considered “nebulization” or “nebulizer therapy”. To our knowledge, there has been no local study examining the journeys of patients with an asthma exacerbation to bronchodilator therapy. We conducted this study to explore the factors contributing to poor asthma control and health-seeking behaviors of patients with asthma.

## Methods

### Design

We explored patients’ journeys from before the start of their symptoms of asthma exacerbation to needing nebulizer therapy, with an emphasis on the delays and factors influencing their self-care decisions prior to seeking medical attention. A qualitative design was chosen to capture patients’ experiences and perspectives.

Ethical approval was obtained from the National Healthcare Group (NHG) Domain Specific Review Board (DSRB). Written informed consent was obtained from all participants.

### Sampling and recruitment

Purposive sampling was used to select patients of different gender, age, ethnicity and duration of asthma attending a polyclinic; to provide variation of experiences from which shared aspects could be distilled.

The inclusion criteria were English-speaking patients aged 21 years old and above with physician-diagnosed asthma who received urgent therapy for an asthma exacerbation at the polyclinic from September 2018 to September 2019. The interviews were conducted within two months of their asthma exacerbation to minimize inaccuracies due to recall bias.

### Data collection

Face-to-face individual semi-structured interviews, each lasting 45–90 min, were conducted by the lead researcher (JSQ), a family physician trained in qualitative research, together with a second or third interviewer. Interviews were audio-recorded digitally. A narrative approach was adopted to explore patients’ experiences, perspectives and concerns without being unnecessarily limited by pre-set questions. The topic guide included questions about asthma history before exploring details of the recent asthma exacerbation, including initial symptoms, perceptions of severity, self-management, help-seeking behavior, symptom progression, and reasons for seeking help at the polyclinic.

### Data analysis

Interview recordings were transcribed verbatim and checked by the lead researcher. Thematic analysis was used. Each transcript was independently coded by at least two researchers, and differences in codes were resolved with the help of a third study team member. NVivo (Version 12) was used to facilitate the coding of transcripts, the analysis of which was undertaken in parallel to identify emerging themes that could be further explored in future interviews. The study team met regularly to discuss coding, identify emerging trends, as well as review and refine the interview topic guide in an iterative process. When 15 individuals had been interviewed, it was agreed that data saturation had been reached with adequate diversity in the participants recruited. This study is reported according to the requirements of the Consolidated Criteria for Reporting Qualitative research (COREQ) checklist [12].

## Results

### Sociodemographic characteristics

Fifteen participants aged 22 to 62 years old were interviewed. Eight were male, 6 Malay, 5 Chinese, 3 Indian and 1 Eurasian. The time since their initial asthma diagnosis ranged from 2 to 50 years (mean 23.9 years) (Table 1).

**Table 1:** Characteristics of participants

Characteristics	Number (%)
*Gender*	
Male	8 (53)
Female	7 (47)
*Age group at interview (years)*	
20–29	4 (27)
30–39	2 (13)
40–49	4 (27)
50–59	4 (27)
60–69	1 (7)
*Ethnicity*	
Chinese	5 (33)
Malay	6 (40)
Indian	3 (20)
Eurasian	1 (7)
*Time since diagnosis (years)*	
1–5	2 (13)
6–10	2 (13)
11–20	4 (27)
≥ 21	7 (47)
*Current work status*	
Unemployed	2 (13)
Employed	13 (87)
Manual	5 (33)
Non-manual	4 (27)
Professional	4 (27)

### Qualitative findings

The journey to nebulization was complex. Four stages could be recognized in patients’ transition from symptom onset to nebulization; the journey often began with poor baseline control, progressing to a trial of self-management, before consideration of when to seek clinical advice and finally the trigger to get medical attention.

### Factors contributing to poor baseline control of asthma

Many participants described a baseline of poorly-controlled symptoms, which then developed into an exacerbation needing nebulizer therapy.

It’s about one, two months after my prednisolone, then it [asthma exacerbation] come again. [...] all along I have the symptoms, so [...] I came [to polyclinic].

(P03, 48/Chinese/Male)

Participants discussed various barriers to asthma control, with costs, either for treatment and review or of medications, emerging as one of the most common challenges.

I got a lot of [...] outstanding bills at polyclinic [...]. So I [...] refuse to go there.

(P01, 24/Malay/Female)

Participants cited multiple reasons for poor adherence to inhalers, some intentional and others unintentional. The latter, like forgetting to administer inhalers, was in the minority. Intentional non-adherence was found to dominate, due to inhaler side effects (e.g. throat discomfort, weight gain) and finding inhaler use troublesome or time-consuming. A few participants perceived no value in using a preventer. Some were concerned about their side effects (e.g. organ injury), not wanting to ‘depend’ on preventers, the sick image created by inhaler use, and not wanting to use inhalers in front of colleagues.

It was like a nightmare [...] using the preventer because I hate the taste of it, and I have to gargle [...]. It’s such a hassle. And having a sore throat.

(P04, 22/Malay/Male)

Several respondents had difficulties attending regular medical follow-ups and ensuring continuity of their preventer supply. This was due to their inability to take time off work and the limited opening hours of polyclinics.

No off day! I [work] Monday to Saturday. Sunday only is mine. How can the polyclinic open? [...] If Sunday can, I can come. [Laughs]

(P05, 58/Malay/Male)

Others alluded to stigma as a barrier to control, verbalizing concerns that publicly acknowledging their asthma would adversely impact their employment, thus limiting their healthcare access.

...working ah, I never say I got asthma. [...] When we ask for work, you put asthma you cannot get the job. [...] That's why ah I seldom go to [...] my company doctor. [...] More I go to the [...] polyclinic.

(P08, 52/Malay/Male)

### Self-management measures

Upon experiencing asthma symptoms, participants engaged in self-management measures as an adjunct to asthma medications before seeking medical attention. They included over-the-counter medications, physical measures and relaxation techniques.

Almost all participants used their reliever medication at the onset of worsening asthma symptoms, but few made adjustments to their preventer regime. Two participants who were previously non-adherent to preventer inhalers started using them (P02 and P07). Two stepped up their preventer (P14 and P15), of which only one made an adequate adjustment by doubling the dosage (P15).

Only one participant spontaneously mentioned the AAP (P15). When the others were asked about such management plans, three had heard of it (P03, P04 and P07), but only one knew what it entailed and the need to escalate the preventer during an exacerbation. Despite this knowledge, she chose not to do so as she perceived her regular dose was already high (P07). None of the participants mentioned taking systemic steroids in response to their asthma symptoms.

I always don’t increase my medication. Even though doctor given me the […] letter will say, first you do this [...] amount, then [...] increase to this amount. [...] If didn’t work, you use the tablets. [...] I usually don’t increase because I know already my inhaler is high dose.

(P07, 34/Indian/Female)

Besides asthma medications, participants used over-the-counter medications for symptom relief, including cough syrup and paracetamol, among others (e.g. lozenges, phlegm medications, cough drops). Five participants used complementary and alternative medicines [Traditional Chinese Medicine, Jamu (traditional Indonesian medicine), Ayurveda, or naturopathy etc.] in their asthma management.

I go search in the Google […] and then, I use the blue ginger. […] I just boil it and drink. I feel better. Go to natural things la, because I don't want depend on medication.

(P14, 62/Chinese/Female)

Most participants tried some form of non-pharmacological measures, which included drinking water, massaging, applying medicated oil, propping themselves up in bed, resting, and employing breathing techniques.

If, for example, I have the attack, I mostly like drinking the hot water first, then after that, I lay down about 5 minutes, then I take my medicine.

(P01, 24/Malay/Female)

Family members and friends provided some of these sources of support, both physical (e.g. slow down respiratory rate, boiling water, massage) and emotional support (e.g. joking with patient).

Sometimes my husband is the one [...] taking care of me. [...] He ask me not to breathe [...] fast, just like slowly take in-and-out breaths.

(P01, 24/Malay/Female)

Adopting strategies to self-regulate their own emotions were often described. These included praying, distraction and breathing exercises.

I will use [...] breathing techniques to calm me down.

(P07, 34/Indian/Female)

### Considerations about when to seek clinical advice

As symptoms persisted or worsened despite self-management measures, participants would consider whether to seek care. They discussed the triggers to action together with the deterrents.

All participants described some form of delay between incomplete response to self-management and eventually seeking treatment at the polyclinic. This delay lasted anywhere from hours to 2 weeks. Six reasons for such delay were identified.

Firstly, many participants cited the inability to access polyclinic out of hours, but 24-h facilities such as hospital A&E, tended not to be used until there was no alternative.

I will just choose [...] polyclinic instead of A&E, unless [...] I can't [manage] [...]. Even the puff, whatever I take, no use, [...] no more breath, [...] then I will go A&E, no choice.

(P09, 49/Chinese/Male)

Many participants expressed strong preferences to attend the polyclinic, justified by low cost, proximity and convenience, and a wish to avoid hospital admission. Polyclinics were perceived to be better equipped than GP clinics and offer shorter waiting times than A&E.

Polyclinic is more convenient [be]cause it's nearer, and [...] the waiting time is relatively lesser, and relatively less costly.

(P10, 28/Indian/Female)

Secondly, participants mentioned a conflict between responding to worsening asthma symptoms and competing priorities like work and family responsibilities. Some participants deferred seeking medical attention until a non-work day, so as not to jeopardize their income.

…normally, I come Saturday, because my working time [...] one Saturday working, one Saturday no working. So I utilize the Saturday la, if not [...] they will cut money.

(P13, 58/Chinese/Male)

One participant shared about the need to prioritize limited finances, choosing to forego urgent asthma care in favor of children’s education.

Because I have financial problems, [...] now with my husband inside [the prison]. [...] The money [...] with me is actually [...] for my [...] eldest son for the school pocket money. [...] The school fees for my two youngests’ nursery is quite expensive. [...] My sickness is important; my kids’ school is [more] important actually. That’s why [...] I [...] refuse going to the doctor.

(P01, 24/Malay/Female)

Thirdly, some participants failed to recognize the onset of an exacerbation.

[...]Because I was thinking is just common cold symptoms.

(P11, 24/Eurasian/Male)

Fourthly, participants did not realize the seriousness of their symptoms.

I felt it wasn't so much of an emergency.

(P10, 28/Indian/Female)

Fifthly, some participants were too physically or mentally exhausted to seek care, perhaps due to asthma or to other events in their lives.

I was very [...] physically exhausted to go and get it checked out the night itself, so I decided to rest, then go the next day.

(P10, 28/Indian/Female)

That time I [...] was [feeling] down [...]. Maybe I was thinking of ending my life also [...]. So I-I never even bother to [...] take the medication [...]. I was literally very, very, very, very down.

(P07, 34/Indian/Female)

Lastly, some participants mentioned a mentality to “tough it out”.

…I thought I am strong enough [laugh] to like just chill and not exert too much physical activities. Yes. Wait it out, then before I go to the doctor.

(P11, 24/Eurasian/Male)

### The trigger to get medical attention

The ultimate trigger to obtain medical attention could be internal (e.g. ineffectiveness of medication, duration of symptoms or impact on everyday life) or an external prompt from someone they knew.

The need for repeated medication with no accompanying improvement prompted some to seek help.

That night [...] 6 [or] 8 times, [...] each time I take 2 [salbutamol puffs]. So like every 2 hour [...] That's why I go to Polyclinic in the morning.

(P12, 41/Malay/Male)

Some were triggered by the duration of troublesome symptoms.

I decide to [...] go for the treatment. [...] I feel like it's about more than two weeks already ah, been feeling the [...] tightness of chest.”

(P11, 24/Eurasian/Male)

The associated disturbance of sleep prompted some,

I suddenly woke up and start coughing a lot. [...] It was affecting my sleep. So I got worried. So I just decided to come the next morning.

(P10, 28/Indian/Female)

while others were prompted to seek medical attention as their symptoms interfered with their ability to work.

I feel very pain [in] my [...] chest ah. All very pain. I want to go work also I felt un-uncomfortable. So [...] I come to uh polyclinic.

(P05, 58/Malay/Male)

The catalyst to seek help sometimes came from a partner, family member or friends who advised the patient to seek medical attention. This interaction could be coercive, or encouraging and helpful.

…my husband keep nagging [...], “I ask you to go doctor then you don’t wanna go.” Then uh, he keep on nagging, then I-I also get mad [...], I scolded him [...]. I we-went to my mum’s house [...], my dad already also nagging “You already got asthma, you don’t wanna go doctor [...]” [...] My husband said, “sleeping beside you ah, can hear wheezing [...]” So the next morning, [...] I went up to the poly[clinic].

(P01, 24/Malay/Female)

On the day itself, [...] I was free and my mom was going to the doctor, I'm like “Why not follow her?”.

(P11, 24/Eurasian/Male)

## Discussion

### Summary of findings

Participants described a background of poorly-controlled asthma even prior to the exacerbation episode, identifying barriers to optimal day-to-day asthma care like cost, underuse of preventer medications, difficulties attending routine asthma care due to work and stigma limiting healthcare access. During an asthma exacerbation, other than using reliever medication, participants often initiated self-management measures, including over-the-counter medications and non-pharmacological interventions like massage and drinking water. Of the 15 patients interviewed, only one stepped up preventer inhaler adequately, as instructed in their AAP. Six reasons for the delay in seeking urgent medical attention during an exacerbation were identified: inability to access polyclinic after-hours, competing priorities in life, difficulty recognizing symptoms of asthma exacerbation, perception that the exacerbation was ‘not serious enough’, being tired or despondent, and a mentality to ‘tough it out’. Factors prompting participants to eventually seek care can be intrinsic (e.g. failure of reliever inhalers, duration of symptoms, sleep disturbance, and inability to work) or extrinsic (e.g. advice from others).

### Comparison with existing literature

#### Factors contributing to poor baseline control of asthma

Patients with poorly-controlled symptoms are more likely to experience asthma exacerbations [13]. Conversely, good symptom control reduces exacerbation risks [7]. It is unsurprising that in this cohort of participants that presented with exacerbations requiring nebulizer therapy, many reported a baseline of poor asthma control. Barriers to optimal asthma control were similarly brought up in other studies, including cost [14–17], non-adherence to ICS [18] due to patient concerns over their safety and fears of dependence [19–22], stigma surrounding inhaler use [23] and inadequate access to healthcare [20, 24]. Interestingly, South Asian participants of another study have also cited concerns that acknowledging their asthma diagnosis would hurt their employment prospects [25], a worry not frequently encountered in the published literature in western populations.

#### Self-management strategies

When asthma symptoms arose, most participants mentioned using non-pharmacological measures like drinking water, resting or lying down, massage, and praying before, or instead of, taking reliever medication, a finding consistent with other studies [15, 17, 20, 26]. Most respondents who had their reliever inhaler on hand reported eventually using it, but it was used acutely to achieve symptom relief rather than as part of their AAP. Disturbingly, most participants were unaware of their AAP and either made no or inadequate changes to their preventer regime despite recognizing symptoms of an asthma exacerbation. While there is no local study explicitly investigating the prevalence of AAP prescription, our findings echo another qualitative study from Singapore, which in 2009 found variable use of AAP among primary care physicians [27].

#### Reasons for delay in seeking care during an asthma exacerbation

We could not find any published local studies focusing on the reasons for the delay in seeking urgent care for asthma. A few studies conducted in western countries similarly found minimization or uncertainty [28] about asthma symptoms, the desire to ‘tough it out alone’ [28, 29], and prioritization of childcare responsibilities over asthma symptoms [30] as factors contributing to the delay. Our study adds to the literature that locally, inability to access polyclinic after-hours, fear of adverse impact on employment, and being too tired or despondent were also contributing factors. Underlying patients’ preference to attend polyclinics over A&E or GP clinics, this study similarly found that important patient considerations were cost [28, 31] and wish to avoid emergency departments [28, 29, 32] and hospitalization. This study additionally found that patients also prize proximity, convenience and availability of asthma care facilities.

#### Triggers for seeking care

AAPs encourage patients to seek medical attention if they experience severe symptoms, or if repeated reliever use does not result in adequate relief. Our study concurs with previous research that the decision to seek urgent care for asthma is not as simplistic [32]. While lack of response to self-management or reliever [32, 33] and prompting by friends or family [32, 33] were also cited in other studies, our study found that patients additionally used duration of symptoms, as well as interference with sleep and work as signposts to need for medical attention. This supports previous research findings that patients’ motivations to seek asthma care differ from health professionals’, with patients driven by socially-influenced factors like limitation of valued activities while practitioners strive for guideline-based ‘control’ of asthma [34].

### Implications for clinical practice

This study has highlighted multiple areas where asthma care can be improved in Singapore.

Firstly, our research has highlighted multiple reasons why patients have poorly-controlled asthma resulting in exacerbations. Recognizing that patients have myriad barriers to asthma control, self-management measures, reasons for delay and triggers to seeking medical attention, clinicians should personalize asthma care and counseling. This includes understanding patients' asthma care goals, or lack thereof [34]. For example, when counseling a patient facing financial constraints, providing him with a breakdown of the respective costs involved in routine asthma care, nebulizer therapy at polyclinics, emergency treatment at A&Es, and intensive care unit (ICU) admission, could help illustrate the cost-effectiveness of adherence to preventer and follow-ups, over allowing exacerbations to occur. This could be done by physicians or asthma nurses, using illustrative cost comparison tables which could also include the various available governmental subsidies and eligibility criteria. While clinic workflows recommend patient reviews one to two weeks after an asthma exacerbation, appointments for these follow-up visits should take into account patients’ schedules to minimize disruptions and reduce barriers to care. A multi-disciplinary team involving nurses, pharmacists, social workers, psychologists, and community partners can better address deeper issues that certain patients face. For instance, patients with financial or social issues would benefit from a social worker’s assistance.

Secondly, the use of AAPs and patient engagement to improve asthma self-management should be optimized. Our study found low knowledge of AAP among patients. Primary care providers should actively engage patients with asthma on self-management skills, especially in identifying symptoms of an asthma exacerbation, appropriate self-care measures and when to seek urgent medical attention. For instance, physicians could point out that during an exacerbation, the use of SABA via a spacer is preferred over air or oxygen-driven nebulization, as it leads to similar improvement in lung function [35], and is more cost-effective [36].

Thirdly, we must improve access to after-hours care. After-hours care options should be included in AAP counseling. Polyclinics could collaborate with nearby GP clinics which are equipped with asthma care facilities. A collated list of these clinics with their respective addresses, opening hours, and approximate costs of after-hours asthma care can be shared with patients.

Issues like healthcare costs and asthma-related stigma are better addressed by health and employment policies. Reducing both direct costs (of consultation and medications) and indirect costs (including loss of earnings and impact on employment prospects) of asthma would reduce barriers to healthcare. For example, moving towards an accountable care organization model may incentivize healthcare providers to focus efforts on prevention (rather than treatment) of asthma exacerbations, thereby achieving cost savings for the healthcare provider and individual patients.

### Strengths and limitations

This paper is unique in examining patients’ journeys to nebulizer therapy in a primary care clinic in Singapore. It explores their difficulties, and when and how they respond to worsening asthma symptoms. The individual interviews conducted with an open-ended approach allowed participants to freely share their thoughts and intimate personal details (e.g. spouse’s incarceration), giving insight into how various facets of patients’ lives contributed to their journeys to nebulizer therapy. This study has highlighted the areas of cost, AAP, after-hours care and holistic management of the patient with asthma, as potential to be addressed to improve asthma care.

This study has several limitations. It was performed on a sample of patients attending a single primary care clinic in Singapore, potentially limiting the generalizability of findings. Although including only English-speaking patients might be a limitation, we managed to recruit a diverse sample comprising all four major ethnicities in Singapore. Given the limitation in accuracy of physician-diagnosed asthma, future studies should consider including only patients with diagnosis of asthma confirmed by pulmonary function tests. Finally, given the sampling frame, we did not track the journeys of asthma patients who sought emergency care from other healthcare facilities.

### Areas for further exploration

This study has identified several areas that require further investigation.

Patients who attend a primary care clinic in need of nebulizer therapy appear reluctant to consider alternatives despite experiencing uncomfortable symptoms. Further study is required to explore these patient preferences and how patients can be empowered to seek care in a timelier fashion.

While many studies, including ours, have highlighted the need for better asthma education to patients and their family and friends involved in their asthma care, the optimal form and method remain unknown. The question of how to promote close ones’ positive influences on patients while minimizing negative impact, also remains unanswered.

The lack of awareness of AAP amongst participants raises the questions of how frequently and how effectively physicians discuss AAPs with patients. While there was a previous local study published in 2009 about family physicians’ perceptions of AAP [27], it would also be useful to re-examine how sentiments and practices have evolved.

Finally, the interventions proposed in this paper (e.g. a polyclinic-GP collaborative model and asthma cost analysis counseling) could be the subject of further research.

## Conclusion

Patients who present to primary care requiring nebulizer therapy for their asthma have complex and unique journeys. A model of care that is responsive to their individual needs via a personalized, multi-disciplinary and collaborative approach holds promise in addressing some of the issues identified. Patients with asthma would further benefit from a broader review of healthcare and employment policies regarding asthma.

## Data Availability

The datasets used and/or analysed during the current study are available from the corresponding author on reasonable request.
